# The Development of Integration in Marsupial and Placental Limbs

**DOI:** 10.1093/iob/oby013

**Published:** 2019-02-08

**Authors:** E M Kelly, J D Marcot, L Selwood, K E Sears

**Affiliations:** 1 School of Integrative Biology, University of Illinois, Urbana, IL61801, USA; 2 Department of Ecology and Evolutionary Biology, University of California at Los Angeles, Los Angeles, CA90095, USA; 3 Department of Zoology, University of Melbourne, Melbourne, Australia; 4 Department of Neurobiology and Behavior, Cornell University, Ithaca, NY 14853, USA

## Abstract

The morphological interdependence of traits, or their integration, is commonly thought to influence their evolution. As such, study of morphological integration and the factors responsible for its generation form an important branch of the field of morphological evolution. However, most research to date on post-cranial morphological integration has focused on adult patterns of integration. This study investigates patterns of correlation (i.e., morphological integration) among skeletal elements of the fore- and hind limbs of developing marsupial and placental mammals. The goals of this study are to establish how patterns of limb integration vary over development in marsupials and placentals, and identify factors that are likely responsible for their generation. Our results indicate that although the overall pattern of correlation among limb elements is consistent with adult integration throughout mammalian development, correlations vary at the level of the individual element and stage. As a result, the relative integration among fore- and hind limb elements varies dynamically between stages during development in both marsupial and placental mammals. Therefore, adult integration studies of the limbs may not be indicative of developmental integration. Results are also consistent with integration during early limb development being more heavily influenced by genetic and developmental factors, and later by function. Additionally, results are generally consistent with a constraint on marsupial forelimb evolution caused by the functional requirements of the crawl to the teat that operates by limiting morphological variation before and at the time of birth, and not after.

## Introduction

The evolution of morphology through the process of natural selection is dependent upon the existence and distribution of morphological variation among individuals. Our understanding of the processes shaping morphological evolution is therefore incomplete without an appreciation of the distribution of this variation, and the factors generating that distribution. In this study, we establish the distributions of morphological variation in the developing limbs of marsupial and placental mammals, and use our results to form hypotheses about the factors (i.e., development, genetics, function) responsible for their generation.

The distribution of morphological variation among phenotypic traits is commonly analyzed in terms of integration, which is defined as the interdependence (i.e., covariance or correlation) of two or more traits (Olson and Miller 1958; Raff 1996; von Dassow and Munro 1999; Gilbert and Bolker 2001; Wagner et al. 2007; Willmore et al. 2007; Goswami and Polly 2010). Morphological integration commonly is thought to significantly impact the evolution of morphology, although the degree to which integration constrains or facilitates morphological evolution remains a point of discussion (i.e., Wagner 1996; Polly 1998; Marriog and Cheverud 2001; Lawler 2008; Polly 2008; Goswami and Polly 2010; Kelly and Sears 2011b; Goswami et al. 2014; Hu et al. 2016; Sherratt et al. 2017).

Much research on morphological integration has focused on characterizing the covariation of traits among adults, and, within adults, among cranial structures (e.g., Cheverud 1982; Cheverud et al. 1983; Cheverud 1988; Marriog and Cheverud 2001; Klingenberg and Leamy 2001; Klingenberg et al. 2001; Ehrich et al. 2003; Zelditch et al. 2004; Marriog and Cheverud 2005; Willmore et al. 2006; Drake and Klingenberg 2008; Marriog et al. 2009; Collar et al. 2014). Less attention has generally been paid to characterizing patterns of morphological integration during development (see, however, Zelditch et al. 2004; Ackermann 2005; Willmore et al. 2006; Zelditch et al. 2006; Mitteroecker and Bookstein 2009; Goswami et al. 2012), or in post-cranial structures (i.e., girdles, limbs, vertebrae) (see, however, Young 2003; Young 2004; Young and Hallgrímsson 2005; Young 2006; Schmidt and Fischer 2009; Young et al. 2010; Bennett and Goswami 2011; Kelly and Sears 2011b; Sears et al. 2012; Fabre et al. 2014; Martín-Serra et al. 2014; Garland et al. 2017; Hanot et al. 2017; Randau and Goswami 2017; Botton-Divet et al. 2018; Hanot et al. 2018; Jones et al. 2018). This represents a fundamental gap in our knowledge, as the evolution of mammalian post-cranial structures has been integral to the diversification of the group into a variety of behavioral, locomotor, and dietary niches (Sears 2004; Polly 2007; Kelly and Sears 2011a). This is definitely the case for marsupial and placental mammals.

Marsupial limbs are less morphologically diverse (i.e., less disparate) than those of placentals (Lillegraven 1975; Cooper and Steppan 2010; Kelly and Sears 2011a), likely as a result of their characteristic mode of reproduction. In contrast to placentals, marsupials give birth after relatively short gestation times to highly altricial young that crawl to the teat under the power of their robust forelimbs (Sharman 1973; Tyndale-Biscoe 1973; Lillegraven 1975; Tyndale-Biscoe and Renfree 1987; Hughes and Hall 1988; Renfree 1993; Shaw and Renfree 2006). The hind limb of the marsupial neonate is relatively undeveloped, and hangs passively from the body during the crawl. Several recent studies suggest that the selective pressures imposed by the functional requirements of the crawl on the marsupial forelimb reduced the genetic and developmental integration, among both marsupial fore- and hind limbs (Doroba and Sears 2010; Kelly and Sears 2011b; Sears et al. 2012; Sears et al. 2012), enabling further specialization of the marsupial forelimbs for the crawl. An outcome of this specialization is that marsupials have to form a specific morphology (i.e., that required for the crawl) at a specific point in time (i.e., birth). This has been shown to effectively constrain the morphological variation of the marsupial forelimb relative to that of placentals, and thereby the evolution of marsupials in general (Sears 2004; Cooper and Steppan 2010; Kelly and Sears 2011a). The lower covariance between homologous elements of adult fore- and hind limbs (i.e., between humerus and femur) in marsupial than placental mammals (Bennett and Goswami 2011; Kelly and Sears 2011) has been cited as evidence for this constraint. However, the developmental origins of these adult patterns of limb integration remain unknown. If the lower level of integration between the fore- and hind limbs of adult marsupials is the result of selective pressures imposed by the crawl, then the reduced integration among these limb elements should be apparent in the skeleton of neonates.

In this study, we investigate the developmental origins of the adult patterns of limb integration in two marsupials (*Monodelphis domestica*, *Trichosurus vulpecula*) and one placental (*Mus musculus*). To do this, we gather limb morphometric data from multiple developmental stages for these species. We use the resulting data to test two related hypotheses: (1) whether the patterns of integration that characterize the fore- and hind limbs of adult marsupials and placentals match the patterns of integration in developing animals, and (2) patterns of fore- and hind limb integration are consistent throughout ontogeny in marsupial and placental mammals. Support for these hypotheses would suggest that patterns of adult limb integration are established by genetic, developmental, and/or functional factors acting early in ontogeny. This finding would also suggest that the reduced integration of adult marsupial limbs is the result of selective pressures imposed by the marsupial newborn’s crawl to the teat. If, however, results of this study conflict with these hypotheses, then this would suggest that patterns of integration vary throughout ontogeny. This finding would be consistent with the contribution of embryonic genetic and developmental factors to adult patterns of limb integration being limited, and inconsistent with the marsupial newborn’s crawl influencing the pattern of limb integration in adult marsupials.

## Materials and methods

### Samples and data

We analyzed three species in this study: the marsupial gray short-tailed opossum (*M.domestica*; terrestrial quadruped), the marsupial common brushtail possum (*T.vulpecula*; arboreal quadruped), and the placental lab mouse (*M.musculus*; terrestrial quadruped). We collected embryos of *M. domestica* (Southwest Biomedical Foundation) and *M. musculus* (ICR Strain, Taconic) from breeding colonies housed within the Sears Lab (University of Illinois). We obtained embryos for *T. vulpecula* from breeding colonies housed in the Selwood lab (University of Melbourne). The embryos were fixed in 95% ethanol and stored at 4°C before use.

We obtained tissues from three stages of *M. musculus* (embryonic day [E] 13.5, *n* = 21; E15.5, *n* = 22; and E17.5, *n* = 21), three stages of *M. domestica* (Stage 33, *n* = 24; post-natal day [PND] 1, *n* = 7; and PND 5, *n* = 6), and two stages of *T. vulpecula* (PND 2, *n* = 6; and PND 18, *n* = 6). In *M. domestica*, Stage 33 occurs shortly before birth (McCrady 1938; Mate et al. 1994). Fewer samples were generally available for *M. domestica* and *T. vulpecula* than *M. musculus* because of the higher difficulty in obtaining these tissues (e.g., breeding colonies having fewer individuals, fewer overall breeding colonies, less breeding in the colonies, etc.), especially of *T. vulpecula*. When possible, we deliberately selected the earliest developmental stages at which each element could be visualized and later stages of limb development for comparison. Later stages of limb development (i.e., immediately after birth and a few days thereafter) were selected to match the ontogeny of *M. domestica* to provide insights into the possible marsupial constraint. We then attempted to select stages of individual species’ ontogeny for which the degree of limb development was roughly comparable to the selected *M. domestica* stages (Wanek et al. 1989) based on the amount of time before weaning, although embryos from *T. vulpecula* were limited in availability. For *T. vulpecula*, PND 2 corresponds to *M. domestica* PND 1 and *T. vulpecula* PND 18 to *M. domestica* PND 5. Embryos and young were eviscerated and skinned before being cleared and stained with alcian blue to visualize cartilage and alizarin red to visualize bone (Hanken and Wassersug 1981). Stained limbs were disarticulated from the rest of the skeleton, and placed with their dorsal sides up.

**Fig. 1:**
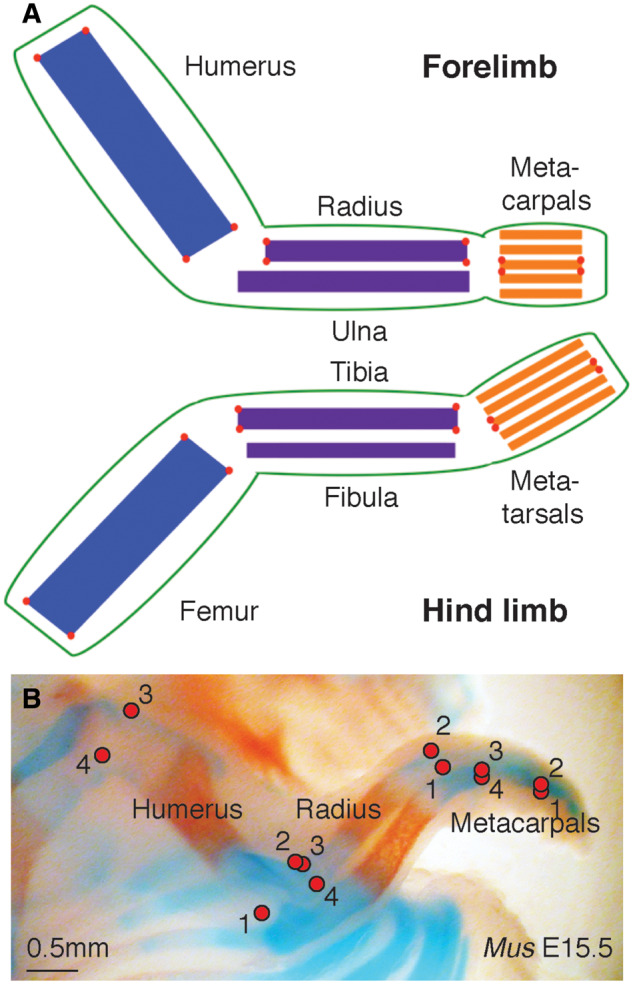
(**A**) Stylized limbs (forelimb on top, hind limb on bottom) depicting the landmarks used in this study. The stylopod is represented in the forelimb by the humerus, and in the hind limb by the femur, the zeugopod is represented in the forelimb by the radius and ulna, and in the hind limb by the tibia and fibula, and the autopod is represented in the forelimb by the metacarpals, and in the hind limb by the metatarsals. (**B**) Cleared and stained mouse E15.5 forelimb. Note that the measurements used in this study were taken directly on specimens using a Reflex Microscope, and not on photos in which landmarks can be more difficult to identify. In both (A) and (B), landmarks are indicated with circles.

We collected three-dimensional coordinates of four landmarks on each stylopod (humerus and femur), zeugopod (radius and tibia), and autopod (metacarpal III and metatarsal III) skeletal element using a Reflex Microscope (for landmark placement, see Fig. 1). We selected these landmarks to capture the length and width of each skeletal element.

### Analyses

Correlation matrices among limb measurements were used as the primary means of analyzing patterns of integration. As a result, correlation matrices for limb elements at all developmental stages were calculated and compared. Before correlation matrices were calculated, a Procrustes analysis was used to scale limb elements to a unit centroid size (Rohlf 1990). The four landmarks of each limb element (i.e., humerus, femur, etc.) were then converted into four linear measurements (the medial and lateral length, and the proximal and distal width of the element). We calculated correlation matrices of log-transformed linear measurements among individuals at each developmental stage of each species separately. Our Procrustes registration normalized configurations to unit centroid size, and thereby distorted the variances of the linear measurements, so we used Pearson product-moment correlation. To assess the repeatability of our correlation matrices (i.e., matrix repeatability), individuals in the limb data set were resampled and the correlation matrix recalculated 10,000 times, and the mean correlation between the original and resampled matrices calculated (Young and Hallgrímsson 2005).

### Comparing overall patterns of covariance throughout ontogeny

To test our second hypothesis (i.e., that patterns of integration are consistent throughout ontogeny), we used pairwise Mantel’s tests to assess how the overall similarity of correlation patterns among limb elements varied among developmental stages and species. Specifically, we used Mantel’s tests to assess the similarity of correlation matrices across developmental stages separately for each species following Marriog and Cheverud (2001) and Mantel (1967). We performed two sets of Mantel’s tests; one including all fore- and hind limb elements, and another using only forelimb measurements. We performed the forelimb-only analyses because most hind limb skeletal elements (i.e., all except the femur) were not sufficiently developed to allow analysis at the earliest marsupial stages (i.e., *M. domestica*, Stage 33).

### Comparing within- and between-limb correlation throughout ontogeny

To further test our second hypothesis, and test our first hypothesis (i.e., adult patterns of limb integration are established by birth), we compared the patterns of correlation of skeletal elements within limbs (i.e., humerus and radius) to the correlation of homologous skeletal elements between limbs (i.e., humerus and femur) throughout development using Mann–Whitney *U*-tests (Sokal and Rohlf 1995; Kelly and Sears 2011b). In these tests, we compared correlation coefficients between pairs of linear measurements within (lengths of the humerus and radius) and between limbs (i.e., lengths of the humerus and femur) for each developmental stage for each species. Among the hind limb skeletal elements of Stage 33 *M. domestica*, only the femur was sufficiently developed to allow reliable quantification. Comparisons among homologous limb elements therefore were limited to the humerus and femur for *M. domestica* at developmental Stage 33. Mann–Whitney *U*-tests were also used to assess the significance of correlations between individual limb elements for each developmental stage for each species.

All analyses were performed in using scripts written in R (http://www.R-project.org 2011) that are available upon request. All raw data from this study are available in the Dryad Digital Repository (datadryad.org).

## Results

### Sampling did not bias developmental correlation matrices

Matrix repeatability was moderately high for all species for all developmental stages (range of 0.81–0.97). This suggests that the sampling restrictions caused by working on limited embryonic materials did not significantly bias the estimation of correlation matrices (*T. vulpecula*, *n* = 6, PND2 = 0.82; *T. vulpecula*, *n* = 6, PND18 = 0.82; *M. musculus*, *n* = 21, E13.5 = 0.88; *M. musculus*, *n* = 22, E15.5 = 0.87; *M. musculus*, *n* = 21, E17.5 = 0.87; *M. domestica*, *n* = 24, Stage 33 = 0.97; *M. domestica*, *n* = 7, PND1 = 0.96; *M. domestica*, *n* = 6, PND5 = 0.94).

### Correlation matrices for most developmental stages are significantly correlated within species

**Fig. 2:**
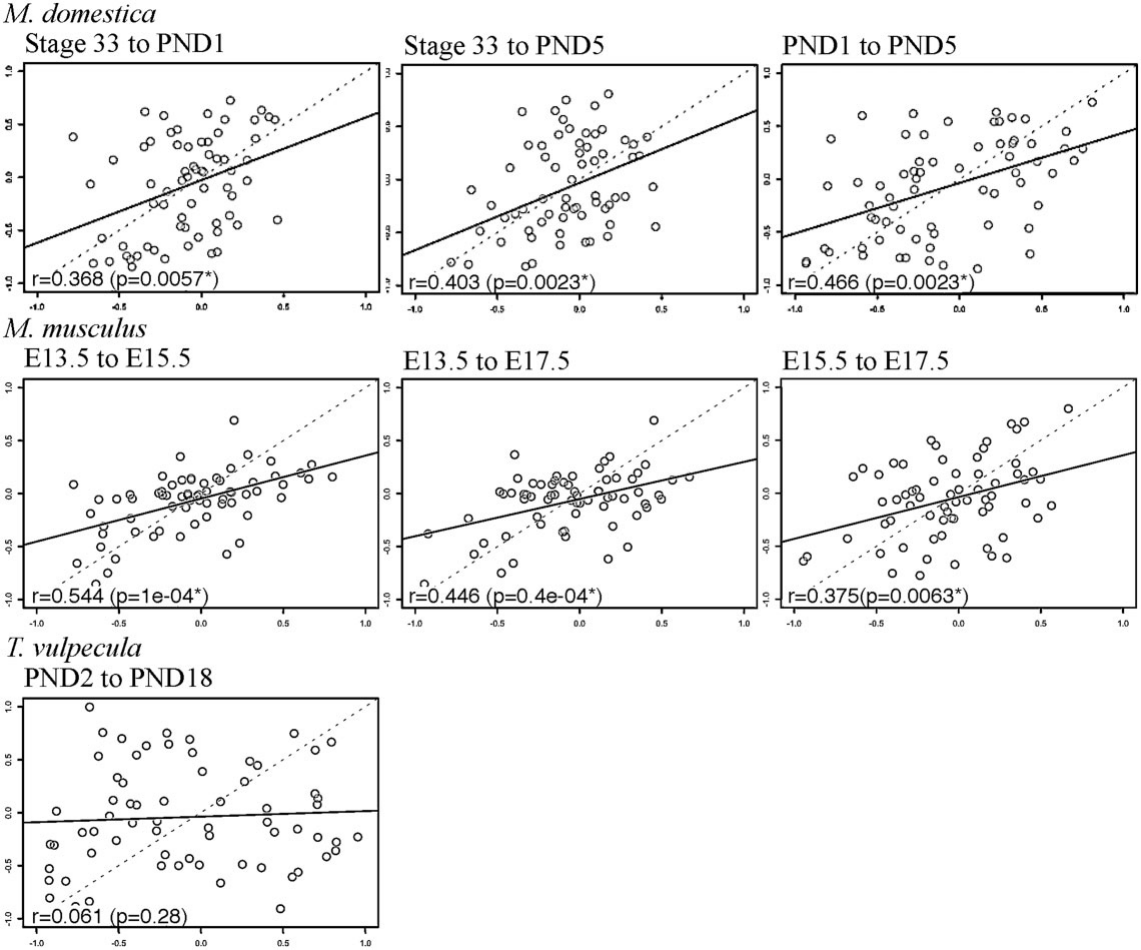
Correlations of the forelimb-only correlation matrices for each developmental stage. *r* = Mantel’s correlation coefficient. Significant *P*-values <0.05 are indicated with an asterisk.

**Fig. 3:**
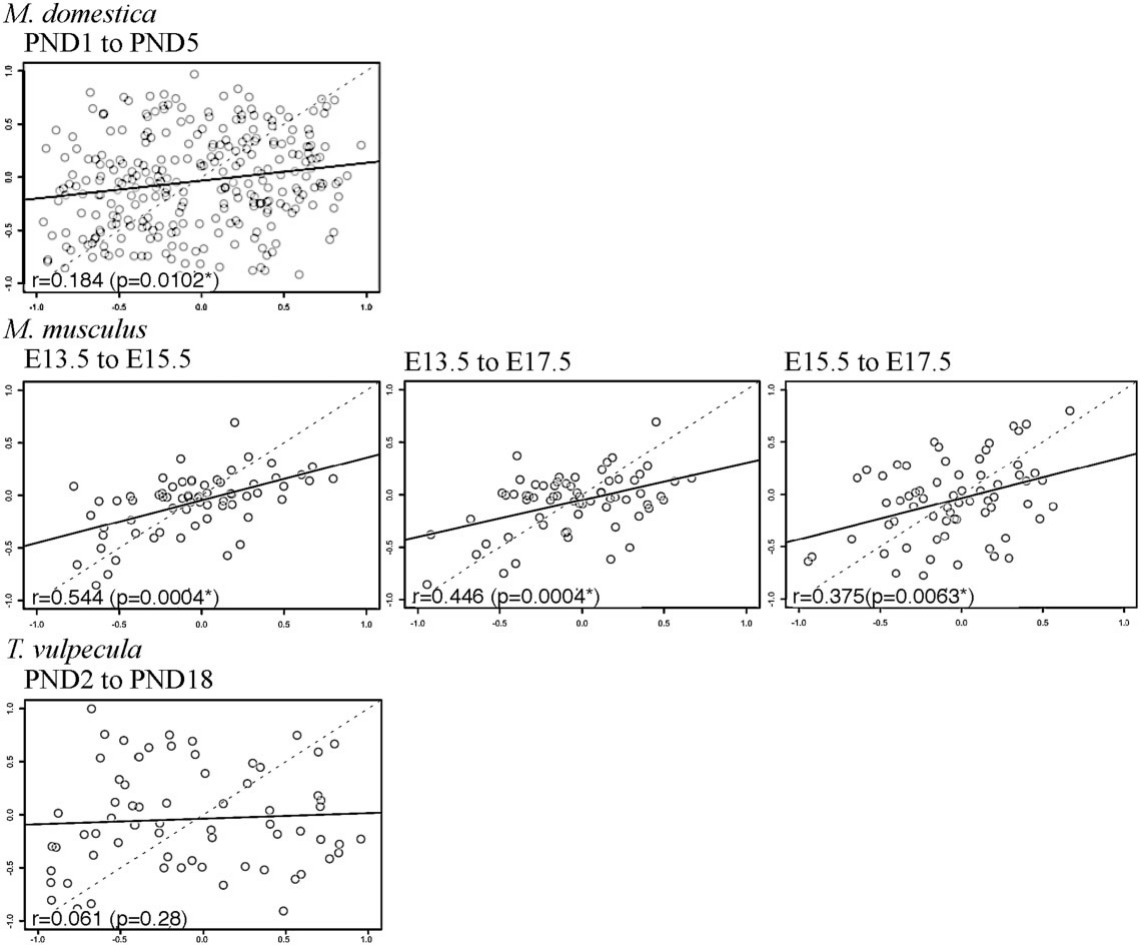
Correlations of the combined fore- and hind limb correlation matrices for each developmental stage. *r* = Mantel’s correlation coefficient. Significant *P*-values <0.05 are indicated with an asterisk.

The similarity of correlation matrices across developmental stages was assessed for each species using Mantel’s tests (Mantel 1967). Pairwise matrix correlation coefficients between all pairs of developmental stages from single species are significantly >0.0 for both the forelimb-only and fore- and hind limb datasets (Figs. 2 and 3), with the exception of the forelimb only matrices of *T. vulpecula* at PND2 and PND18 (*r* = 0.061, *P *=* *0.280). In most cases, correlations of the forelimb-only datasets (*M. domestica*—Stage 33 to PND1, *r* = 0.368, *P *=* *0.006; Stage 33 to PND5, *r* = 0.403, *P *=* *0.002; PND1 to PND5, *r* = 0.466, *P *=* *0.002; *M. musculus*—E13.5 to E15.5, *r* = 0.544, *P *<* *0.001; E13.5 to E17.5, *r* = 0.466, *P *<* *0.001; E15.5 to E17.5, *r* = 0.375, *P *=* *0.006) are higher than those of the combined fore- and hind limb dataset (*M. domestica*—PND1 to PND5, *r* = 0.184, *P *=* *0.010; *M. musculus*—E13.5 to E15.5, *r* = 0.206, *P *<* *0.001; E13.5 to E17.5, *r* = 0.207, *P *=* *0.001; E15.5 to E17.5, *r* = 0.221, *P *<* *0.001). The exception to this is *T. vulpecula*, where the correlation was stronger with hind limb data included (PND2 to PND18, *r* = 0.242, *P *<* *0.001).

### Correlation coefficients of most limb elements do not significantly differ through ontogeny

The significance of the similarity between the correlation coefficients among measurements of individual limb elements at each developmental stage was assessed with a series of Mann–Whitney *U*-tests (Sokal and Rohlf 1995). This assessment was performed separately for each species. Results of these tests indicated that correlation coefficients do not significantly differ between most limb elements (Table 1). However, correlation coefficients do significantly differ between the skeletal elements of the fore- and hind limb (i.e., between the humerus and femur, radius and femur, metacarpal and femur) of *M. domestica* individuals shortly before birth (Stage 33). Correlation coefficients also significantly differ between and the metacarpal and tibia of *M. musculus* individuals at E13.5.

**Table 1:** Pairwise comparisons of correlation coefficients for limb elements

	Humerus	Radius	Metacarpal	Femur	Tibia	Metatarsal
*M. domestica*, Stage 33						
Humerus	NA	25.000	27.000	5.000	NA	NA
Radius	0.310	NA	19.000	4.000	NA	NA
Metacarpal	0.180	0.937	NA	3.000	NA	NA
Femur	0.041*	0.026*	0.015*	NA	NA	NA
Tibia	NA	NA	NA	NA	NA	NA
Metatarsal	NA	NA	NA	NA	NA	NA
*M. domestica*, PND1						
Humerus	NA	16.000	19.000	14.000	18.000	30.000
Radius	0.818	NA	23.000	20.000	18.000	27.000
Metacarpal	0.937	0.485	NA	15.000	17.000	26.000
Femur	0.589	0.818	0.699	NA	19.000	26.000
Tibia	1.000	1.000	0.937	0.937	NA	28.000
Metatarsal	0.065	0.180	0.240	0.240	0.132	NA
*M. domestica*, PND5						
Humerus	NA	15.000	11.000	16.000	18.000	8.000
Radius	0.699	NA	15.000	19.000	19.000	10.000
Metacarpal	0.310	0.699	NA	23.000	22.000	15.000
Femur	0.818	0.937	0.485	NA	19.000	10.000
Tibia	1.000	0.937	0.589	0.937	NA	10.000
Metatarsal	0.132	0.240	0.699	0.240	0.240	NA
*M. musculus*, E13.5						
Humerus	NA	17.000	28.000	15.000	9.000	19.000
Radius	0.937	NA	29.000	13.000	13.000	19.000
Metacarpal	0.132	0.093	NA	6.000	5.000	7.000
Femur	0.699	0.485	0.065	NA	14.000	23.000
Tibia	0.180	0.485	0.041*	0.589	NA	27.000
Metatarsal	0.937	0.937	0.093	0.485	0.180	NA
*M. musculus*, E15.5						
Humerus	NA	7.000	19.000	6.000	21.000	16.000
Radius	0.093	NA	26.000	8.000	23.000	19.000
Metacarpal	0.937	0.240	NA	12.000	23.000	19.000
Femur	0.065	0.132	0.394	NA	30.000	28.000
Tibia	0.699	0.485	0.485	0.065	NA	9.000
Metatarsal	0.818	0.937	0.937	0.132	0.180	NA
*M. musculus*, E17.5						
Humerus	NA	7.000	25.000	20.000	16.000	18.000
Radius	0.093	NA	25.000	28.000	24.000	26.000
Metacarpal	0.310	0.310	NA	13.000	10.000	14.000
Femur	0.818	0.132	0.485	NA	12.000	17.000
Tibia	0.818	0.394	0.240	0.394	NA	21.000
Metatarsal	1.000	0.240	0.589	0.937	0.699	NA
*T. vulpecula*, PND2						
Humerus	NA	13.000	25.000	19.000	15.000	15.000
Radius	0.485	NA	26.000	20.000	22.000	20.000
Metacarpal	0.310	0.240	NA	10.000	10.000	10.000
Femur	0.937	0.818	0.240	NA	17.000	18.000
Tibia	0.699	0.589	0.240	0.937	NA	17.000
Metatarsal	0.699	0.818	0.240	1.000	0.937	NA
*T. vulpecula*, PND17						
Humerus	NA	28.000	21.000	29.000	12.000	17.000
Radius	0.132	NA	9.000	16.000	8.000	9.000
Metacarpal	0.699	0.180	NA	28.000	15.000	15.000
Femur	0.093	0.818	0.132	NA	6.000	7.000
Tibia	0.394	0.132	0.699	0.065	NA	18.000
Metatarsal	0.937	0.180	0.699	0.093	1.000	NA

*Notes*: The upper-right section of the each table contains the Mann–Whitney *U* values and the lower-left the associated *P*-values. Significantly different correlation coefficients (*P *<* *0.05) are in bold and marked with an asterisk.

NA = comparisons for which data from at least one limb element were not available.

### Correlation coefficients of skeletal elements within limbs are higher than those of homologous elements between limbs at the oldest developmental stages

Means of the absolute values of the correlation coefficients among skeletal elements were calculated for the forelimbs, hind limbs, and homologous elements of the fore- and hind limbs (Table 2). Ratios of correlation coefficients for skeletal elements within (i.e., humerus and radius) and between (i.e., humerus and femur) limbs were also calculated, and the significance of differences in the distributions of correlation coefficients within and between limbs were determined using Mann–Whitney *U*-tests (Table 2). Correlation coefficients are greater for within than between limb comparisons for *M. domestica* (PND5), *M. musculus* (E13.5, E15.5, and E17.5), and *T. vulpecula* (PND18). However, the distributions of correlation coefficients within and between limbs only significantly differed for the oldest examined stages of *M. domestica* (PND5) and *M. musculus* (E17.5).

**Table 2:** Mean correlation coefficients among limb elements (calculated using absolute values)

	Within forelimb	Within hind limb	Between fore- and hind limb	Ratio of within to between	Pooled within vs. between, *P*-value
*M. domestica*, Stage 33	0.349	NA	NA	NA	NA
*M. domestica*, PND1	0.383	0.363	0.378	0.988	0.682
*M. domestica*, PND5	0.435	0.455	0.340	**1.309**	0.001*
*M. musculus*, E13.5	0.257	0.366	0.285	**1.093**	0.322
*M. musculus*, E15.5	0.377	0.356	0.352	**1.041**	0.866
*M. musculus*, E17.5	0.224	0.239	0.155	**1.492**	0.001*
*T. vulpecula*, PND2	0.588	0.488	0.547	0.982	0.825
*T. vulpecula*, PND18	0.388	0.552	0.415	**1.133**	0.114

*Notes*: Within forelimb includes the correlations between the humerus, radius, and metacarpals, and within hind limb the correlations between the femur, tibia, and metatarsals. Between fore- and hind limbs includes the correlations between homologous elements of the fore- and hind limbs—the humerus and femur, radius and tibia, and metacarpals and metatarsals. The ratio of “within to between” is the ratio of the correlation coefficients for the within limb comparisons (i.e., within forelimb and within hind limb) to those of the between fore- and hind limb comparisons. Ratios >1 (highlighted in bold) indicate that average correlation coefficients are higher within than between limbs. In “pooled within vs. between,” the *P*-value is calculated from a statistical comparison of the correlations among serially homologous elements (i.e., humerus to femur) to the correlations among elements from the same limb (i.e., humerus to radius) using the Mann–Whitney *U*-test. Statistically significant results (*P *<* *0.05) are in bold and indicated with an asterisk.

NA = comparisons for which data were not available.

## Discussion

Results of the matrix correlation analyses suggest that overall patterns of correlation among skeletal elements of the fore- and hind limbs are highly similar between the developmental stages examined in this study. This finding is generally consistent with our second hypothesis, that patterns of integration are stable throughout ontogeny. These results are also consistent with those of previous studies on mammalian cranial variation (e.g., Zelditch and Carmichael 1989b; Willmore et al. 2006). However, inconsistent with our first hypothesis, this study also identified several specific ways in which patterns of correlation vary among developmental stages by analyzing correlations among individual skeletal elements.

Our results in *M. musculus*, the species for which our sample size is largest, suggest that correlation coefficients between elements of the same limb increase relative to those of homologous elements in different limbs throughout the stages of development included in this study. These results provide evidence that patterns of integration vary through ontogeny, and contradict our first and second hypotheses. Thus, though commonly assumed to be predictive, adult integration studies may not be indicative of developmental integration. This finding is also consistent with the genetic regulation of early development (and therefore early patterns of morphological integration) being shared in placental fore- and hind limbs as a result of their shared evolutionary history as serial homologues (Young and Hallgrímsson 2005), and with functional factors increasing in their relative importance to limb integration throughout ontogeny (Zelditch et al. 1992). In line with function playing a role in changing patterns of limb correlations in mouse, mouse limbs first begin to move around E14 and E15, between our first two developmental time points (Kodama and Sekiguchi 1984).

In contrast to the patterns observed in *M. musculus*, this study found that correlation coefficients significantly differed between skeletal elements of the fore- and hind limbs (specifically, between the humerus and femur, radius and femur, metacarpal and femur) of *M. domestica* immediately before birth (Stage 33) but were similar at later developmental stages (i.e., PND1 and PND5). These later patterns of developing integration in *M. domestica* (PND1 and PND5) are not consistent with patterns of integration in adult *M. domestica* (Kelly and Sears 2011a). Furthermore, results suggest that correlation coefficients between elements of the same limb (i.e., humerus and radius) tend to decrease (PND1) and then increase again (PND5) relative to those of homologous elements in different limbs (i.e., humerus and femur) in *M. domestica*. Correlation coefficients between skeletal elements of the fore- and hind limbs were also similar in *T. vulpecula* at later stages of development (PND2 and PND17), but earlier developmental stages were not available for this species. Taken together, these results are consistent with the lower level of integration among the fore- and hind limbs of adult marsupials being established at the time of the birth (i.e., our first hypothesis), but not with patterns of integration being consistent throughout ontogeny (i.e., our second hypothesis).

The relatively reduced integration between the fore- and hind limbs of the marsupial *M. domestica* immediately before birth and in adulthood may be the result of different genetic, developmental, and/or functional factors (Willmore et al. 2007). Thus, it is unlikely that the newborn’s crawl to the teat constrains marsupial evolution by influencing adult patterns of morphological integration. Instead, these results and those of previous studies (Doroba and Sears 2010; Kelly and Sears 2011a, 2011b; Sears et al. 2012) suggest that it is more likely that the crawl to the teat constrains marsupial limb evolution by influencing limb variation before and at the time of birth. Specifically, results suggest that selection for a forelimb with the ability to complete the crawl reduced the genetic and developmental integration and, thereby, the morphological integration between the fore- and hind limb before and at the time of birth. This reduced integration in turn allowed the forelimb to become more specialized for the crawl. The need to form the specialized crawl morphology at birth limited the morphological variation present in the forelimb before and at the time of birth (Sears 2004; Sears et al. 2012). As natural selection is dependent upon the existence of morphological variation, this in turn constrained the evolution of the marsupial forelimb. However, it is important to note that the variances of the marsupial fore- and hind limbs, as well as of the marsupial and placental forelimbs, from this study are statistically indistinguishable. Furthermore, sample sizes for *M. domestica* and *T. vulpecula* in this study were limited by available tissues, which in turn limited the power of our analyses. Thus, further studies with increased sampling are needed to confirm these results.

In summary, although the overall pattern of correlation among limb skeletal elements is conserved throughout development in the mammalian species included in this study, the relative integration of fore- and hind limb elements varies quite dynamically in most examined species during this time. This finding is consistent with previous studies which found that patterns of integration among cranial skeletal elements are repeatedly re-patterned over the course of mammalian development (Zelditch 1988; Zelditch and Carmichael 1989a, 1989b; Ackermann 2005; Zelditch et al. 2006; Mitteroecker and Bookstein 2009; Goswami et al. 2012). Results of this study also support the hypothesis that developmental and genetic sources of integration dominate earlier in ontogeny, while later integration may more closely reflect functional influences (Zelditch et al. 1992). As a result, findings of this study suggest that adult patterns of limb integration are less likely to be significantly influenced by genetic, developmental, or functional factors acting during early limb morphogenesis, and are more likely to reflect factors impacting growth and variation of limbs after birth.
